# Advantages of Using Paclitaxel in Combination with Oncolytic Adenovirus Utilizing RNA Destabilization Mechanism

**DOI:** 10.3390/cancers12051210

**Published:** 2020-05-12

**Authors:** Elora Hossain, Umma Habiba, Aya Yanagawa-Matsuda, Arefin Alam, Ishraque Ahmed, Mohammad Towfik Alam, Motoaki Yasuda, Fumihiro Higashino

**Affiliations:** 1 Department of Molecular Oncology, Faculty of Dental Medicine and Graduate School of Biomedical Science and Engineering, Hokkaido University, Sapporo 060-8638, Japan; elora@den.hokudai.ac.jp (E.H.); dr.ishraqueahmed@gmail.com (I.A.); 2 Department of Cancer Pathology, Faculty of Medicine, Hokkaido University, Sapporo 060-8638, Japan; habiba@med.hokudai.ac.jp; 3 Department of Vascular Biology and Molecular Pathology, Faculty of Dental Medicine and Graduate School of Dental Medicine, Hokkaido University, Sapporo 060-8586, Japan; ayayana9@den.hokudai.ac.jp (A.Y.-M.); towfik1977@yahoo.com (M.T.A.); 4 Department of Restorative Dentistry, Faculty of Dental Medicine and Graduate School of Dental Medicine, Hokkaido University, Sapporo 060-8586, Japan; arefin@den.hokudai.ac.jp; 5 Department of Oral Molecular Microbiology, Faculty of Dental Medicine and Graduate School of Dental Medicine, Hokkaido University, Sapporo 060-8586, Japan; moyasuda@den.hokudai.ac.jp

**Keywords:** Oncolytic adenovirus, E1A, AU-rich element (ARE), HuR, ARE-mRNA, Paclitaxel, Microtubules, CAR

## Abstract

Oncolytic virotherapy is a novel approach to cancer therapy. Ad-*fos*ARE is a conditionally replicative adenovirus engineered by inserting AU-rich elements (ARE) in the 3’-untranslated region of the E1A gene. In this study, we examined the oncolytic activity of Ad-*fos*ARE and used it in a synergistic combination with the chemotherapeutic agent paclitaxel (PTX) for treating cancer cells. The expression of E1A was high in cancer cells due to stabilized E1A-ARE mRNA. As a result, the efficiency of its replication and cytolytic activity in cancer cells was higher than in normal cells. PTX treatment increased the cytoplasmic HuR relocalization in cancer cells, enhanced viral replication through elevated E1A expression, and upregulated CAR (Coxsackie-adenovirus receptor) required for viral uptake. Furthermore, PTX altered the instability of microtubules by acetylation and detyrosination, which is essential for viral internalization and trafficking to the nucleus. These results indicate that PTX can provide multiple advantages to the efficacy of Ad-fosARE both in vitro and in vivo, and provides a basis for designing novel clinical trials. Thus, this virus has a lot of benefits that are not found in other oncolytic viruses. The virus also has the potential for treating PXT-resistant cancers.

## 1. Introduction

Viral therapy based on conditionally replicating adenoviruses (CRAds) is a promising cancer therapeutic strategy, as these viruses selectively replicate and lyse cancer cells [1,2]. CRAds are obtained by either modifying viral gene by mutation or by the insertion of cancer-specific agents [3]. We developed an adenovirus designated Ad-*fos*ARE by inserting AU-rich elements (ARE) in the 3’-untranslated region (3′-UTR) of the E1A gene.

AREs are usually located in the 3′-UTR of mRNAs and are the best-known determinants of mRNA instability. Studies on the expression of cytokines and proto-oncogenes have shown that many 3′-UTRs contained AREs, and subsequently, some of their translation is controlled by these AREs [4,5,6]. AREs are primarily defined by their ability to promote rapid de-adenylation-dependent mRNA decay [6]. There is relatively lesser sequence similarity among AREs; the *c-fos* mRNA with the most typical ARE contains AUUUA pentamers and/or U-rich sequences [4,7]. AREs control mRNA degradation and translation via interactions with RNA binding proteins, which specifically bind to ARE. mRNAs carrying AREs are considered unstable and are translated only for a short period, as in the case for transcription factors, cytokines, oncogenes, and certain RNA transcripts of DNA viruses [7,8]. Cellular RNA binding proteins, such as Tristetraprolin (TTP), ARE/poly (U)-binding/degradation factor 1 (AUF 1), Hu antigen R (HuR), and T-cell internal antigen-1 (TIA-1), bind to mRNAs that contain AREs. ARE binding proteins (AREBPs) either aim for the mRNA to enter the degradation pathway (TTP and AUF1) [8,9], protect the mRNA from degradation (HuR), or suppress their translation (HuR and TIA) [10]. HuR is a member of the embryonic lethal abnormal vision (ELAV) family of RNA-binding proteins, which is ubiquitously expressed by most cell types. Intracellularly, HuR is localized mainly in the nucleus, but shuttles between the nucleus and the cytoplasm [11,12]. Cytoplasmic HuR protects ARE-mRNAs against rapid degradation by binding with ARE [13]. In normal cells, HuR transiently relocalizes to the cytoplasm under stress conditions. In contrast, in cancer cells, HuR is constitutively localized in the cytoplasm and speculated to be involved in the ARE-mRNAs stabilization and transformation of cancer cells [14,15].

Oncolytic adenovirus can be used either as a standalone therapeutic modality or in combination with other approaches to overcome or enhance viral activity in the tumor microenvironment. Paclitaxel (PTX) is the most common anticancer agent used against many cancers, but the emergence of drug resistance is a major drawback. PTX is a microtubule (MTs) stabilizing drug [16,17] and inhibits cell replication by enhancing the polymerization of tubulin monomers into stabilized microtubule bundles that are unable to reorganize into the proper structures during mitosis [17,18]. MT networks are very important for adenovirus entry, nuclear trafficking, development of replication compartments, and controls broader aspects of infected-cell behavior [19]. After entering through the receptor, the viral capsid interacts with the motor protein dynein and trafficking to the nucleus in a microtubule-dependent manner [20]. Virus infection and consequent signaling alter the MT dynamics of the host cell, thus making MTs stable for the intracellular transport of the virus [21].

PTX can increase the expression of the coxsackie and adenovirus receptor (CAR) [22,23]. Adenoviral fiber knob of the capsid binds with cell surface receptor CAR and initiates gene transfer [24]. CAR is a 46-k transmembrane glycoprotein and belongs to the immunoglobulin superfamily, which is the primary receptor for adenovirus [24,25,26]. Furthermore, PTX increases the HuR export to the cytoplasm in cancer cells and thus facilitates mRNA stabilization [27]. Some studies have shown that the ubiquitously expressed HuR can utilize either the actin- or microtubule-dependent cytoskeleton for the transport of mRNA cargo [28,29,30]. Microtubules are important for cell motility and the transport of organelles, such as vesicles, and are relevant for long-distance transport of protein and mRNA [30,31,32].

In this study, we investigated the efficacy of our developed oncolytic adenovirus Ad-*fos*ARE. Ad-*fos*ARE showed the potential as an oncolytic virus and synergism with a microtubule stabilizer PTX. The use of heat shock or a knockdown depleted HuR, which further down-regulated Ad-*fos*ARE replication, indicated that Ad-*fos*ARE replicates in an ARE-mRNA stabilization-dependent manner. PTX promotes the nuclear export of HuR, which can enhance the propagation of Ad-*fos*ARE in cancer cells but not in normal cells. Furthermore, it up-regulated the expression of CAR and increases virus internalization. The oncolytic activity of Ad-*fos*ARE was enhanced synergistically by PTX through the increase of E1A expression and enhancement of viral apoptotic activity both in vitro and in vivo. These results indicate that PTX offers many benefits to Ad-*fos*ARE.

## 2. Results

### 2.1. Construction of the Virus for Selective Replication in Cancer Cells

**Figure 1:**
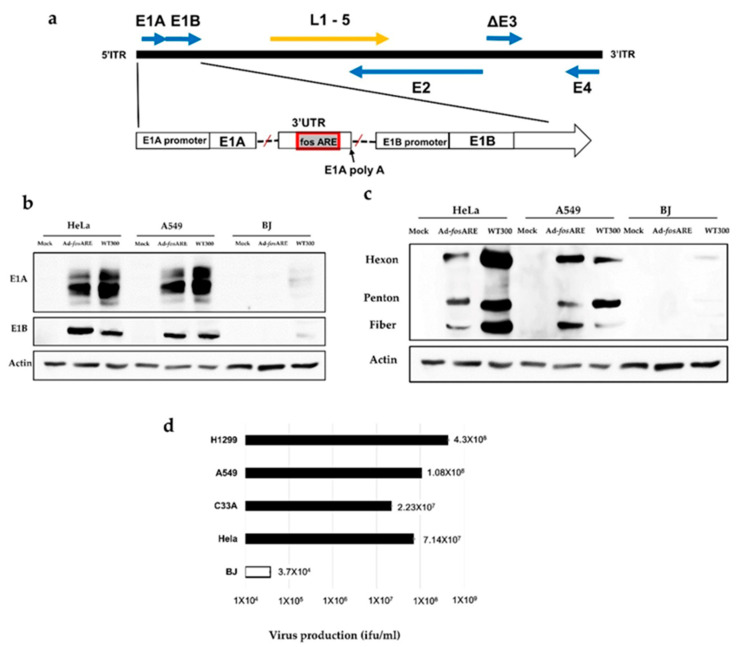
The structure of Ad-*fos*ARE and its production efficiency. (**a**) Schematic representation of Ad-*fos*ARE with *c-fos* ARE in the 3′-UTR of the E1A gene. Arrows indicate the location of early (E1, 2, and 4) and late (L1–5) genes. (**b**) Expression of viral proteins in Ad-*fos*ARE and WT300-infected cells. A549, HeLa, and BJ cells were infected by both viruses and the expression of early proteins E1A, E1B and (**c**) late proteins hexon, penton, and fiber (right) were estimated by western blot analysis. Actin expressions were also assessed as the internal control. (**d**) Cancer (HeLa, C33A, A549, and H1299) cells and normal (BJ) cells were infected with Ad-*fos*ARE at an MOI of 10 ifu/cell and virus production was determined 48 h after infection. Each titer (ifu/ml) is shown on the graph. Data presented here are mean ± standard deviation of three independent experiments.

Ad-*fos*ARE was constructed by inserting ARE of *c-fos* gene into the 3′-UTR of the adenovirus E1A gene (Figure 1a). The *c-fos* ARE causes rapid degradation of E1A mRNA in normal cells, but stabilizes it in cancer cells, due to the presence of HuR in the cancer cell cytoplasm. To examine the expression of E1A (30–50 kDa) protein, two cancer cell lines, HeLa and A549, and normal cells (BJ) were infected with Ad-*fos*ARE at an MOI of 100 (ifu/cell) for 96 h, as viral protein expression of Ad-*fos*ARE started after 72 h (Figure S1). E1A protein levels were high in Ad-*fos*ARE-infected A549 and HeLa cells and low in normal BJ cells (Figure 1b). Wild type adenovirus (WT300) was used as a positive control. We also examined the expression of early gene product E1B55k (Figure 1b) and late viral proteins Hexon, penton, and fiber (Figure 1c) and the observed similar results as E1A, and we detected very little to no protein expression in the normal cells.

To examine the production efficiency of Ad-*fos*ARE, cancer cell lines (HeLa, C33A, A549, and H1299) and normal cells (BJ) were infected at an MOI of 10 ifu/cell and viral titers generated after 48 h were detected by hexon staining was done on cells infected with these viruses. Our results showed that in cancer cells, the propagation of Ad-*fos*ARE was very high (Figure 1d). These results indicate that Ad-*fos*ARE can replicate significantly higher in cancer cells than in normal cells.

**Figure 2:**
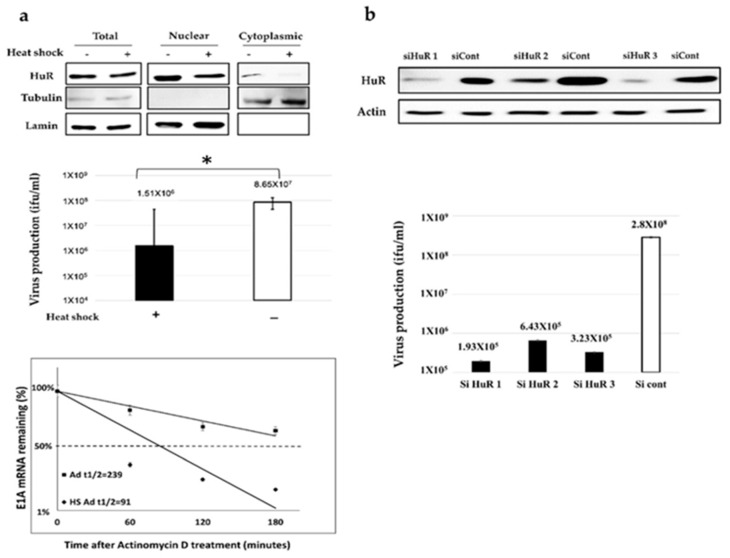
The effects of HuR-depletion on E1A mRNA stabilization and Ad-*fos*ARE replication. (**a**) HeLa cells were given heat-shock at 43 °C for 2 h, and the amount of HuR in total, nuclear, and cytoplasmic fraction were determined using western blot analysis. β-tubulin and lamin expression were used as cytoplasmic and nuclear fraction markers (top). Virus production was assessed, and the values were compared to the non-treated cells (middle) (* indicates *p* < 0.05). HeLa cells were infected with Ad-*fos*ARE (MOI 10 ifu/cell) and heat-shocked immediately after infection at 43 °C for 2 h. Transcriptions were inhibited by actinomycin D 5 µl/mL for 60, 120, and 180 minutes. After the indicated periods, cells were harvested and extracted for total cellular RNA. mRNA levels are quantified by quantitative real-time RT-PCR experiments, using Glyceraldehyde 3-phosphate dehydrogenase (GAPDH) as a normalization control. Graphs depict the percentage of remaining E1A mRNA levels with GAPDH mRNA and compared with the standards of normalized mRNA species measured immediately after the addition of actinomycin D and which were set as 100%. The half-lives of the mRNAs (min) are indicated as t½ (bottom). (**b**) HeLa cells were transfected with a HuR targeting siRNA (HuR 1, HuR 2, and HuR 3) and a negative control siRNA, and the expression of HuR was estimated by western blot analysis. (top) HuR KD-HeLa cells were infected with Ad-*fos*ARE, and viral titers were determined as described in materials and methods after 48 h of virus infection. (bottom) siHuR, siRNA targeting HuR; siCont, control siRNA treated cells. Data shown above presented as mean ± standard deviation of three independent experiments.

To investigate whether Ad-*fos*ARE replicates in an ARE-mRNA stabilization-dependent manner, Ad-*fosARE* replication was examined under conditions, which either decreased or enhanced ARE-mRNA stability. To evaluate virus production in HuR-depleted cells, where ARE-mRNA cannot be stabilized, we treated cells with heat shock (HS). HS treatment of HeLa cells for 2 h resulted in reduced expression of HuR protein in the cytoplasm of cells and also shortened the half-life of E1A mRNA containing ARE (Figure 2a bottom). Likewise, HS-treated cells showed a significant reduction in virus production compared to the non-treated cells (Figure 2a middle).

Since HS treatment affects many factors other than HuR degradation in cells, we confirmed the HuR-depletion by HuR knockdown (KD) (Figure 2b). We found that virus propagation was decreased in siRNA-treated cells compared to control siRNA-treated cells (Figure 2b). Taken together, these results indicate that HuR is essential for Ad-*fos*ARE replication and it replicates in an ARE-mRNA stabilization-dependent manner. Our previous report showed that HuR depletion (HS and HuR KD) causes the downregulation of the replication of WT300 because HuR is required for full replication of adenovirus [33]. However, since the downregulation rate of WT300 was lower than that of Ad-*fos*ARE, and this is because the dependency of HuR of Ad-*fos*ARE is significantly higher than that of WT300.

### 2.2. In Vitro Cytolytic Potential of Ad-fosARE

**Figure 3:**
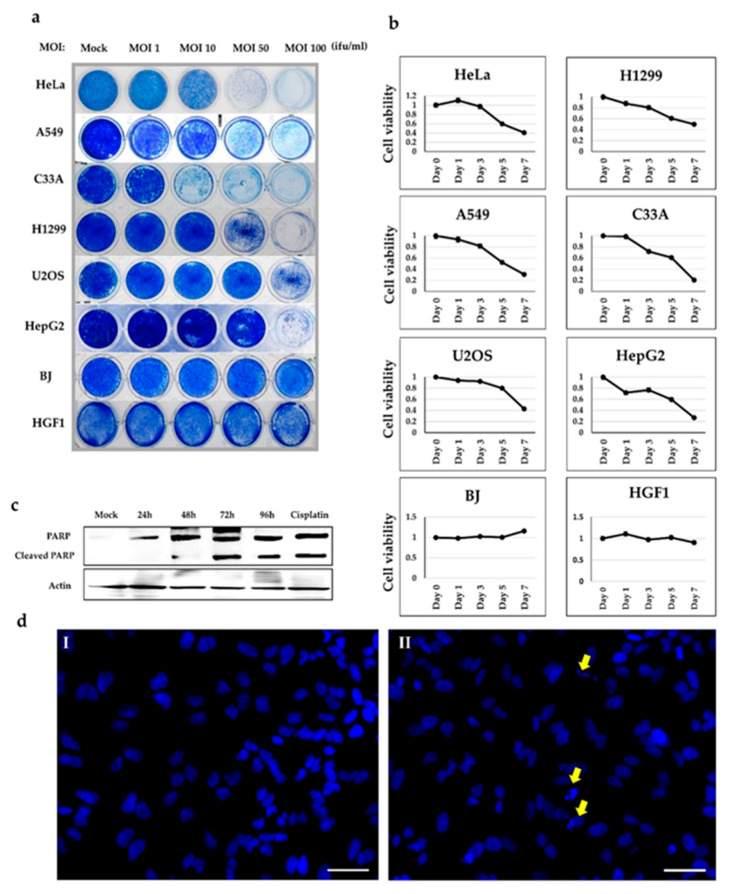
Cell lysis activity of Ad-*fos*ARE. (**a**) Cancer (HeLa, A549, C33A, H1299, U2OS, and HepG2) cells and normal (BJ and HGF1) cells were infected with the virus at MOIs indicated. Cells were stained using Coomassie brilliant blue 7 days post-infection. Living cells stained blue. (**b**) Cell metabolic activities of Ad-*fos*ARE-infected cells were measured to estimate the cell lysis activity using the XTT assay. The same cancer and normal cells were infected with the virus at a MOI of 100 ifu/cell and cell viabilities were estimated 1, 3, 5, and 7 days post infection. (**c**) Cleaved PARP levels were assessed by western blot analysis to detect the cell death activity mediated by Ad-*fos*ARE. PARP expression in cisplatin-treated cells were used as a positive control [34]. (**d**) HeLa cells were infected with Ad-*fos*ARE with MOI 100 ifu/cell. The apoptosis of cells was also analyzed by Hoechst 33,342 staining 96 h after infection. Nuclear fragmentation and chromatin clumping were observed in the virus treated cells (Yellow arrows). Scale bar: 50 µm. Three replicates represented each assay.

To further assess the cell lysis activity of Ad-*fosARE*, we examined cell death in virus-infected cells using a cytopathic effect (CPE) assay. Six different cancer cell lines (HeLa, C33A, A549, H1299, HepG2, and U2OS) and two normal cell lines (BJ and HGF1) were infected with the virus at MOIs of 1, 10, 50, or 100 ifu/cell. Cytotoxicity was assessed by staining the infected cells with Coomassie brilliant blue 7 days after infection (Figure 3a). The virus killed all cancer cells in a dose-dependent manner, while most normal cells survived at all doses. These results showed the in vitro-selective cytolytic activity of Ad-*fos*ARE.

To estimate the cell lysis activity of Ad-*fos*ARE, we examined cell viability using the XTT assay. Cancer cell lines (HeLa, A549, U2OS, H1299, C33A, and HepG2) and normal cells (BJ and HGF1) were infected with Ad-*fos*ARE at an MOI of 100 (ifu/cell), and the XTT assay was performed 1, 3, 5, and 7 days after infection (Figure 3b). Our results showed that while most cancer cells died after 7 days in a time-dependent manner, most normal cells infected with the virus remained viable at the same time point.

Next, we explored the pathway behind Ad-*fos*ARE induced cell death in cancer cells. As various pathways of adenovirus-induced cell death such as apoptosis, autophagy have been reported [3,34], we examined the proteins expressed specifically for each cell death. Although LC3, expressed autophagy, was not detected in Ad-*fos*ARE infected cells (Erola Hossain, Hokkaido University, Spporo, Japan, personal communication, 2020), only the processing of the caspase substrate PARP was detected by western blot analysis on various days post-infection (Figure 3c). We confirmed the apoptosis of the cells using Hoechst 33,324 staining to observe the morphological changes due to cell apoptosis in Ad-*fos*ARE infected cells. Morphological changes, such as condensation of chromatin and nuclear fragmentations, were observed clearly in the Ad-*fos*ARE infected cells (Figure 3d). These results suggest that Ad-*fos*ARE infection-induced apoptosis in the cancer cells.

### 2.3. Comparison of the Oncolytic Effects of Ad-fosARE and dl1520

**Figure 4:**
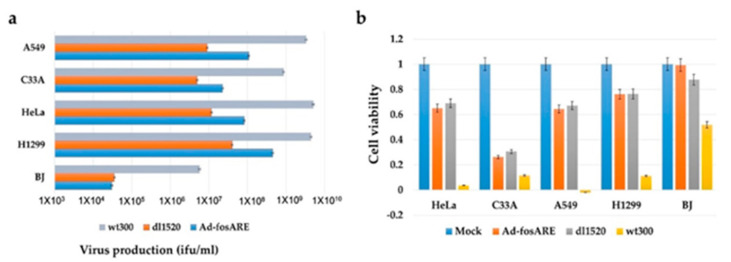
Comparison of the virus production and cell lysis activity of Ad-*fos*ARE, dl1520 and WT300. (**a**) Cancer cells (HeLa, C33A, A549, and H1299) and normal cells (BJ) were infected with Ad-*fos*ARE, WT300, and dl1520 at MOI of 10 ifu/cell. Virus production was assessed 48 h after infection. Each titer (ifu/ml) is shown on the graph. (**b**) Cell viabilities were estimated 7 days post-infection by XTT assay. Data presented here are mean ± standard deviations of three independent experiments.

Since an E1B-55k gene deleted-adenovirus dl1520 equivalent to H101, which is clinically applied [35,36], we compared the oncolytic activity and viral replication of Ad-*fos*ARE with dl1520 and wild type adenovirus WT300. Ad-*fos*ARE, dl1520, and WT300 were infected at an MOI of 10 ifu/cell, in cancer cell lines (HeLa, C33A, A549, and H1299) and normal BJ cells, and virus titers were estimated 48 h after infection. As shown in Figure 4a, Ad-*fos*ARE virus production was higher than dl1520 virus production in all cancer cells. Furthermore, we compared the cytolytic activity by XTT assay, as presented in Figure 4b. Cytolytic activity of Ad-*fos*ARE and dl1520 were comparable in cancer cells, and in normal cells, Ad-*fos*ARE caused less damage compared to dl1520.

### 2.4. The Potential Synergistic Activity of Paclitaxel

**Figure 5:**
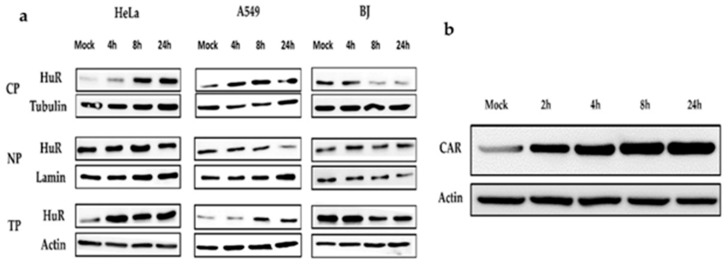
Effects of paclitaxel on cytoplasmic HuR exportation and expression of CAR. (**a**) Cancer cells (HeLa, A549) and normal cells (BJ) were treated with 4 nM paclitaxel (PTX¬). Cell lysates were separated into the cytoplasmic and nuclear fractions, and HuR localization was estimated by western blot analysis. The level of the HuR protein was monitored in total cell lysates (TP), cytoplasmic (CP), and nuclear extracts (NP). Tubulin, Lamin, and actin are used as the internal control for each fraction. (**b**) After treatment with PTX, total HeLa cell lysate was collected, and western blot analysis was performed to detect CAR.

Low concentrations of PTX increase the cytoplasmic exportation of HuR (36 kDa) [27], thus, we examined whether a low dose of PTX (4 nM) could induce the cytoplasmic HuR export. This was done with the speculation that the upregulation of the cytoplasmic HuR expression could further increase the replication of Ad-*fos*ARE. We treated cancer cells with PTX and collected the cytoplasmic and nuclear protein. Furthermore, we performed western blot analysis and observed an increase in cytoplasmic HuR export (Figure 5a). Normal BJ cells treated with PTX showed decreased HuR after 24 h of treatment. 

Coxsackie-adenovirus receptor (CAR), which is the primary affinity receptor for adenovirus, was also upregulated by PTX [22,23]. To check CAR (46 kDa) expression levels, HeLa cells were treated with 4 nM PTX for different periods, and whole-cell lysates were collected for western blot analysis. As shown in Figure 5b, CAR expression increased after treatment with PTX.

### 2.5. Synergistic Effect of Oncolytic Adenovirus and Paclitaxel

**Figure 6:**
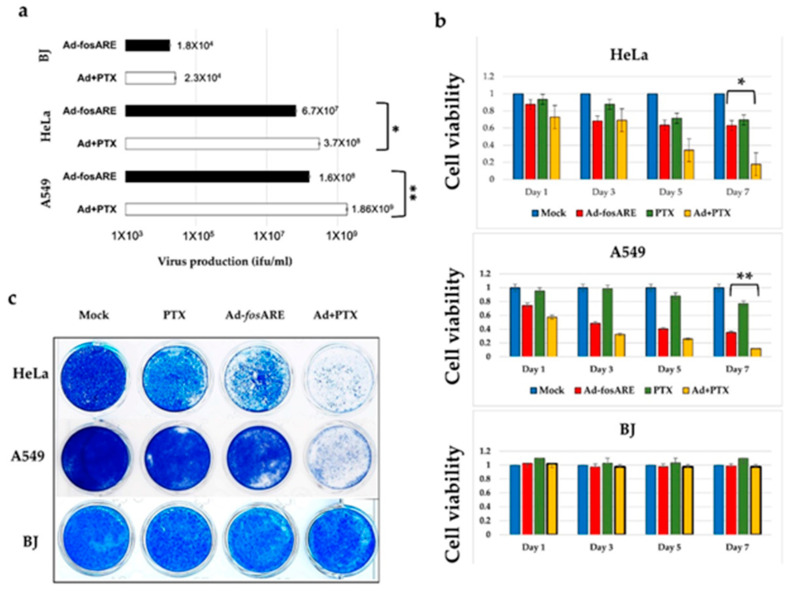
Assessment of combinatorial treatment’s effect (Ad-*fos*ARE and PTX) on viral replication and cell lysis activity. (**a**) Cancer (HeLa and A549) cells and normal (BJ) cells were infected with Ad-*fos*ARE at an MOI of 10 ifu/cell and 4 nM PTX. Virus production was determined 48 h after through Hexon staining. Each titer (ifu/mL) is shown on the graph. Data are shown above as the mean ± standard deviation of three independent experiments. (**b**) Cell lysis activities of combination treatment were measured using the XTT assay. As mentioned above, cancer and normal cells were infected with the virus at an MOI of 10 ifu/cell and cell viabilities were estimated 1, 3, 5, and 7 days after infection. (**c**) Prior mentioned cancer and normal cells were subjected to staining with Coomassie brilliant blue 7 days after infection to observe cytopathic effects. Living cells stained blue. (* indicates *p* < 0.05, ** indicate *p* < 0.01).

To explore the potential relationship between Ad-*fos*ARE replication and chemotherapy, we determined the propagation levels of the virus after combination treatment. HeLa, A549, and BJ cells were treated either with the virus only or with a combination of PTX and virus. Virus titers generated after 48 h were detected by staining for hexon protein. We found that after combination treatment, virus titers increased by five- to 10-fold in cancer cells, but no significant change was observed in normal cells (Figure 6a).

The XTT assay was performed at 1, 3, 5, and 7 days after viral infection in HeLa, A549, and BJ cells (Figure 6b). In the case of HeLa cells, each treatment had little effect until about the third day, but the combined effect with 4 nM PTX and virus (MOI of 10) began to appear from about the fifth day. On the other hand, in A549 cells, PTX alone had little effect, but when used in combination, the effects began to appear from about day 3, and almost all cells died after 7 days. However, in the case of normal BJ cells, no synergistic effect was observed. Although both HeLa and A549 cells showed dose-dependent cytotoxicity after exposure to PTX, A549 cells were slightly more sensitive than HeLa cells. The combination index (CI) for HeLa ranged from 0.5 to 0.85, and for A549 ranged from 0.40 to 0.52, as calculated using Chou-Talalay equations and analyzed by Compusyn (Figure S2). A CI of <0.9 indicates synergism and a CI between 0.9 and 1.1 indicates a potential additive effect, whereas a CI of >1.1 indicates antagonism [37].

Furthermore, we assessed the cytopathic effects of this combinatorial approach. First, we treated the cells with PTX for 4 h and subsequently infected them with the virus. After 7 days post-infection, almost all cancer cells were killed, indicating a cumulative effect (Figure 6c). Significantly, no cytotoxic effect of combination treatment was observed for normal cells. Taken together, these results indicate that PTX can enhance the activities of Ad-*fos*ARE in a synergistic manner.

### 2.6. Effects of Paclitaxel Treatment on Ad-fosARE Viral Protein Synthesis and mRNA Stabilization

**Figure 7:**
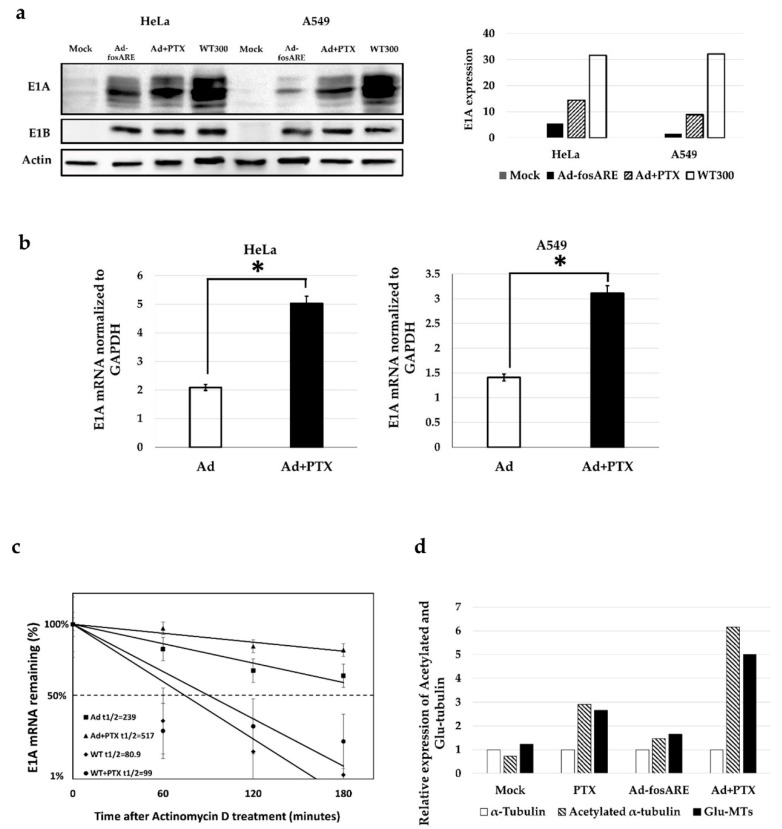
Effects of paclitaxel on Ad-*fos*ARE replication. (**a**) To check the viral protein synthesis, HeLa and A549 cells were treated in combination with Ad-*fos*ARE (MOI 10) and PTX (4 nM). E1A and E1B55kd were estimated by western blot analysis. Densitometric data of E1A expression (left) (**b**) With the same dose of treatment E1A mRNA was detected by quantitative real-time RT-PCR using GAPDH mRNA as a normalized control. (**c**) E1A mRNA stabilization in Ad-*fos*ARE infected cells. HeLa cells were infected with Ad-*fos*ARE and WT300 (MOI-10 ifu/cell) and combination with PTX. Transcriptions were inhibited by actinomycin D 5 µl/mL for 60, 120, and 180 minutes. After the indicated periods, cells were harvested and extracted for total cellular RNA. mRNA levels are quantified by quantitative real-time RT-PCR experiments using GAPDH as a normalization control. Graphs depict the percentage of remaining E1A mRNA levels with GAPDH mRNA and compared with the standards of normalized mRNA species measured immediately after the addition of actinomycin D and which were set as 100%. The half-lives of the mRNAs (min) are indicated as t½. (**d**) To determine post-translationally modified tubulin, HeLa cells were treated with either Ad-*fos*ARE, PTX or a combination of both. The expression of acetylated and Glu-MTs was quantified by densitometry and the values were normalized to non-infected conditions. Data are shown as the mean ± standard deviation of three independent experiments (* indicates *p* < 0.05).

The effect of paclitaxel on viral protein synthesis was assessed by western blot analysis. HeLa and A549 cells were treated for 72 h with either only virus or a combination of the virus at MOI of 10 ifu/cell and PTX (4 nM). Subsequently, total cell lysate was assessed to detect E1A (30–50 kDa) and E1B (~55 kDa) (Figure 7a). Quantification of the western blot results showed significant changes in E1A protein synthesis in cells subjected to combinatorial treatment.

We also examined the E1A mRNA expression by quantitative real-time RT-PCR, and the results were in line with western blot results as the E1A mRNA expression was significantly higher in combination treatment after 72 h of treatment (Figure 7b). The half-life of ARE, including E1A mRNA transcribed from Ad-*fos*ARE was prolonged by PTX treatment from 239 to 517 minutes. On the other hand, E1A mRNA without ARE synthesized by WT300 was changed from 80.9 to 99 minutes (Figure 7c). These data suggest that PTX enhances Ad-*fos*ARE replication through ARE containing E1A mRNA.

### 2.7. Combination Treatment Increases the Level of Post-Translationally Modified Tubulin

Next, we examined whether Ad-*fos*ARE affected paclitaxel activity by upregulating-acetylated α-tubulin, which is the marker for stabilized microtubules [21]. After quantifying the western blot analysis data, it was evident that the level of acetylated α-tubulin increased with combination treatment by 48 h (Figure 7d). Alteration of the MTs dynamic instability is essential for the trafficking of the virus to the nucleus. Virus infection causes modulation of MT dynamics by post-translationally modifying MTs through a RhoA-dependent mechanism. Post-translationally modified MTs are detyrosinated (Glu-MTs) and acetylated MTs. Results show that both Glu-MTs and acetylated α-tubulin was higher in combination treatment (Figure 7c, Figure S3).

### 2.8. Effects of Paclitaxel on the Cell Lysis Activity of the Virus

To determine the combined effect of paclitaxel and virus on cell death, we examined the levels of caspase substrate PARP (116 kDa) and cleaved PARP (89 kDa) (Figure S4). HeLa cells were treated and the whole lysate was collected after 96 h and subjected to western blot analysis. Since both full length and cleaved PARP were found in combination treatment and positive control, PTX enhanced apoptosis of the cancer cells infected with Ad-*fos*ARE.

### 2.9. In Vivo Synergistic Effect of Ad-fosARE and PTX in Murine Flank Tumor

**Figure 8:**
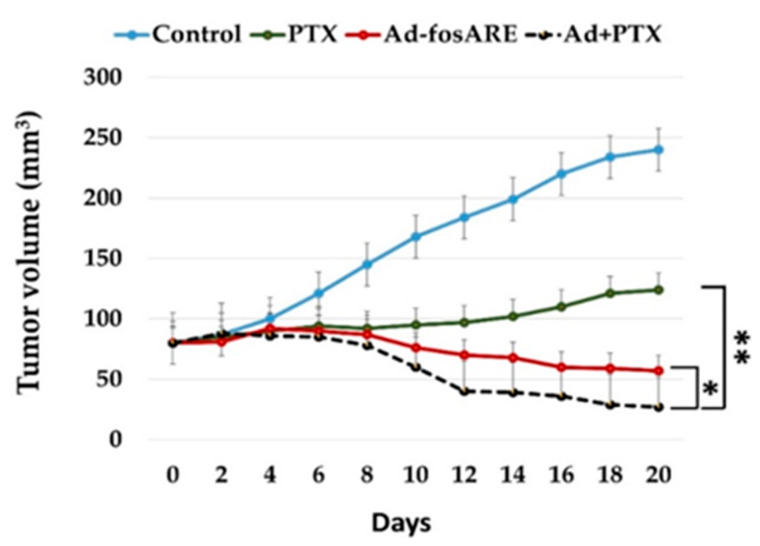
In vivo tumor lysis potential of the Ad-*fos*ARE and PTX combination therapy in human cervical cancer xenograft nude mice. HeLa S3 cells were injected subcutaneously into the flanks of female BALB/C nude mice and allowed to grow to ~56 mm in diameter. PTX (0.4 mg in 100 µL of PBS) or vehicle (cremophor EL/dehydrated ethanol/saline) was given by intraperitoneal (i.p.) injection. 1 × 10^6^ ifu (100 µL) of Ad-*fos*ARE or the same volume of PBS was injected intratumorally (i.t.) thrice (days 1, 4, and 7) directly into the tumors after PTX injection. Tumor volumes were calculated by the following equation: Volume (mm^3^) = A × B^2^ × 0.5 (A is considered the longest diameter; B is considered shortest diameter). * indicates *p* < 0.05, ** indicate *p* < 0.001.

To examine the effects of Ad-*fos*ARE and PTX therapy in vivo, a flank tumor model of human HeLa-derived cervical cancer in five-week-old female BALB/c nu/nu mice was used. Once the tumors reached ~56 mm in diameter, they were treated with the equivalent volume of PBS, Ad-*fos*ARE, PTX or Ad-*fos*ARE+PTX. The combination of Ad-*fos*ARE+PTX showed a significantly reduced mean tumor volume (± 27 mm^3^) compared to Ad-*fos*ARE alone (± 57 mm^3^), PTX alone (± 124 mm^3^), or control (± 240 mm^3^). Tumors treated with the PTX and Ad-*fos*ARE in combination had a smaller mean volume compared to only Ad-*fos*ARE, only PTX, and control (Figure 8). The study was terminated when the tumors of the control group began to ulcerate. No morbidity attributable to either tumor progression or therapy was observed. These results indicate the in vivo synergistic effect of Ad-*fos*ARE and PTX.

## 3. Discussion

Among the currently investigated oncolytic viruses, the use of oncolytic adenovirus strains appears to be the most promising. In the present study, we demonstrated the oncolytic efficacy of the Ad-*fos*ARE with ARE of the *c-fos* gene, in the 3′UTR of the E1A gene. Our results showed that the replication efficiency of this virus in cancer cells was higher than in normal cells; moreover, it was better than the dl1520 equivalent to H101, which is clinically applied [35]. Furthermore, to overcome the limitation of an oncolytic virus as a standalone therapy, the combination of oncolytic virus and the chemotherapeutic agent was investigated. This approach was explored to see whether the two treatment modalities enhanced or synergized the effects of each other. Our results showed that PTX treatment synergistically enhanced the oncolytic activity of Ad-*fos*ARE both in vitro and in vivo. To the best of our knowledge, this is the first study to show that PTX activates oncolytic adenovirus function by promoting HuR cytoplasmic translocation.

We first investigated the requirement of mRNA stabilization system for Ad-*fos*ARE replication. Since Ad-*fos*ARE possesses an ARE downstream of E1A gene, an increased expression level of E1A mRNA was expected in cancer cells, where ARE-mRNA is stabilized, thereby promoting virus growth. To confirm such ARE-mRNA-dependent virus growth and cell lysis, HuR was depleted by heat shock and knockdown to avoid ARE-mRNA exportation and stabilization. As expected, the virus replication decreased in response to inhibited ARE-mRNA stabilization. In contrast, virus replication was induced by PTX treatment, which increased the cytoplasmic HuR translocation (Figure 6a). Additionally, the half-life of E1A mRNA was low in HuR depleted cells compared to control cells. After treatment with PTX, the half-life of E1A mRNA was increased, and these results indicate that Ad-*fos*ARE grows in an ARE-mRNA stabilization-dependent manner, thus increasing the level of cytoplasmic HuR in cancer cells by PTX enhances the oncolytic activity of Ad-*fos*ARE.

Some studies demonstrated that nucleo-cytoplasmic HuR shuttling could utilize either the actin- or microtubules (MT)-dependent cytoskeleton for the transport of mRNA cargo [28,32,38]. In our previous study, we reported that in cancer cells, HuR can translocate to the cytoplasm in an actin-independent manner, but is actin-dependent in normal cells [39]. Although direct interaction between microtubules and HuR has not been demonstrated, it is speculated to be mediated by p38 mitogen-activated (MAP) kinase [32,40]. PTX polymerizes and stabilizes the MT and arrests the cell cycle in G2+M phase and can increase the HuR mediated mRNA stabilization [32,41,42]. In our study, HuR export was induced in cancer cells in the presence of PTX, while it decreased in normal cells, possibly due to the altered MT dynamics. In normal cells, PTX at a concentration of 1 μM directed the cells towards G2+M phase arrest, but in cancer cells, the cytotoxic effect could be observed at a concentration as low as 0.010–0.05 μM [43,44]. In this study, we used 4 nM PTX; thus, the proliferation of Ad-*fos*ARE could be activated more due to the stabilization of ARE-mRNA. In previous studies, the combination of oncolytic virus and PTX were used with a focus on the synergism between mitotic arrest and apoptosis [16,45]. In contrast, in the present study, we focused on cytoplasmic HuR translocation and stabilization of mRNA. This feature is unlikely to be observed in other oncolytic viruses, due to considerable unique advantages of Ad-*fos*ARE. Since many types of cancer cells have been reported to stabilize ARE-mRNA [46,47]. Ad-*fos*ARE may be clinically relevant for the treatment of various cancers.

After adenovirus internalization into the host cells through viral receptors, MTs extending towards cell periphery usually encounter the virus, and the associated dynein motors bind them to MTs. Adenovirus-infection increased the post-translationally modified MTs through upregulated Glu-MTs and acetylated MTs through the RhoA-dependent mechanism, which favors viral delivery to the nucleus [21]. In this study, we showed that PTX upregulated the MT stabilization by utilizing the acetylated α-tubulin. We found that the levels of post-translationally modified MTs were increased after 1 h of treatment with PTX, and the levels were even higher with the combination of Ad-*fos*ARE and PTX. The ability to change the MTs dynamics of the host cells is the other advantage of the combinatorial treatment with PTX. Hence, PTX significantly influences the replication and spread of Ad-*fos*ARE, and additionally, the virus may also enhance the antitumor activity of PTX through the modification of MTs.

In conclusion, in most of the cancer cells, HuR relocates to the cytoplasm and stabilizes ARE-mRNA [32]. For Ad-*fos*ARE replication, cytoplasmic HuR relocalization and E1A-ARE-mRNA stabilization are necessary. In contrast, in the normal cell, where there is no cytoplasmic HuR, E1A-ARE-mRNA was degraded due to existence of ARE and the viral replication was inhibited. In the present study, we have shown that PTX synergizes with Ad-*fos*ARE by increasing the cytoplasmic HuR export, and, as shown previously, upregulating CAR and altering MTs dynamic instability. These findings indicate that PTX aids this oncolytic adenovirus engineered with ARE in multiple ways.

## 4. Materials and Methods

### 4.1. Cell Lines

A549 and H1299 (human lung cancer cell lines), HeLa, HeLa S3, C-33 A (human cervical carcinoma cell lines), 293 (human embryonal kidney cells transformed by the adenovirus E1 gene), U-2 OS (human bone osteosarcoma cell line), HepG2 (human liver cancer cell line), HGF1 (human gingival fibroblast cell line), and BJ (human foreskin fibroblast cell line) were used in this study. Cells were acquired from the American Type Culture Collection (ATCC; Manassas, VA, USA) and cultured in Dulbecco’s modified Eagle’s medium (DMEM; Sigma-Aldrich; Merck KGaA, Darmstadt, Germany) including 10% fetal bovine serum (FBS; Biowest, Nuaille, France) with antibiotics at 37 °C in a 5% CO_2_ atmosphere in humidified conditions.

### 4.2. Construction of Ad-fosARE

Ad-*fos*ARE was constructed using a pXhoIC plasmid and pAdenoX-PRLS (Clontech, Inc., Mountain View, CA, USA). pXhoIC has the E1 region of the type 5 human adenovirus genome. A 69-base pair of synthesized ARE fragment (5′- TTTTATTGTG TTTTTAATTT ATTTATTAAG ATGGATTCTC AGATATTTAT ATTTTTATTT TATTTTTTT -3′) of the *c-fos* gene was inserted into the HpaI site in the 3′-UTR of the E1A gene of pXhoIC. A fragment of the E1 region was then amplified by PCR, and inserted into the deleted E1 region of the adenovirus genome in the pAdenoX-PRLS by in-fusion technique (Fw: 5′-GTAACTATAACGGTCATTTGTCTAGGGCCGCGGGGACTT-3′, Rv: 5′-ATTACCTCTTTCTCCGCCACGCCCACACATTTCAGTACC-3′). The entire genome of Ad-*fos*ARE was isolated by cutting using PacI and the fragment was then transfected into 293 cells. Virus particles were concentrated by several rounds of viral infection and cells were collected in order to prepare a virus lysate by subjecting them to three cycles of freezing and thawing. The titers of the infectious unit (ifu) of Ad-*fos*ARE were determined using the Adeno-XTM Rapid Titer Kit (Clontech) and 293 cells according to the manufacturer’s instructions.

Wild-type adenovirus type 5 (WT300) (generous gift from Dr T. Shenk; Princeton University, NJ, USA), E1B55k-deleted mutant adenovirus (dl1520) (generous gift from Dr A.J. Berk; University of California, Los Angeles, CA, USA) were used in this study.

### 4.3. Drug, Reagents, and Antibodies

Paclitaxel (PTX; Sigma Aldrich Co., St. Louise, MO, USA) was diluted in DMSO (Tissue culture grade; Sigma Aldrich Co., St. Louise, MO, USA) and added to the medium for in vitro study. For in vivo experiments PTX was dissolved in cremphor EL (EMD Millipore Corp, Darmstadt, Germany) and dehydrated ethanol (1:1) to a concentration of 50 mg/ml and stored at −20 °C. Before injection, PTX was diluted in 0.9% saline to a final concentration of 4 mg/ml for in vivo studies. Actinomycin D (from Streptomyces species) were purchased from Sigma-Aldrich (USA).

For western blot analysis, the following antibodies were used: antibody specific to E1A (M58, sc-58658, Santa Cruz Biotechnology, Dallas, TX, USA), EIB-55k (2A6, generous gift from T. Shenk), Adenovirus type5 late gene products (L133, generous gift from T Dobner), PARP (46D11 9523, Cell Signaling Technology, Danvers, MA, USA), Actin (Actin(C4) hrp, sc- 1616: Santa Cruz Biotechnology, Dallas, TX, USA), HuR (3A2, sc-5261; Santa Cruz Biotechnology, OR, USA), Lamin B (C-20, sc-6216, Santa Cruz Biotechnology, Dallas, TX, USA), β-tubulin (05-661; EMD Millipore Corp., Darmstadt, Germany), CAR (E1-1, sc-56892, Santa Cruz Biotechnology, Dallas, TX, USA), anti-Tubulin Detyrosinated (cat. no. 05-661; Millipore Corp., Darmstadt, Germany), and anti-acetyl- α tubulin (05-661; Millipore Corp. Darmstadt, Germany) as primary antibodies. Anti-mouse (m-IgGκ BP-HRP: sc-516102, Santa Cruz Biotechnology, Dallas, TX, USA) and anti-rabbit (mouse anti-rabbit IgG-HRP: sc-2357, Santa Cruz Biotechnology, Dallas, TX, USA) as secondary antibodies.

Antibody bands were visualized using SuperSignal West Femto Maximum Sensitivity Substrate (Thermo Fisher Scientific, Inc., Waltham, MA, USA).

### 4.4. Immunocytochemistry

Cells were grown on coverslips in 35 mm dishes at 60–80% confluence and treated with virus and drug for Hoechst 33,342 staining for detection of cell death. After treatment, cells were rinsed once with phosphate buffer saline (PBS), fixed for 20 min at RT with 4% paraformaldehyde in PBS, and blocked and permeabilized in 2% BSA plus 0.1% Triton X-100 in PBS at room temperature. Cell nuclei were stained with Hoechst 33,342 before mounted on slides by using Mountant permafluor (Thermo scientific, FM 111212A, Waltham, MA, USA). Cells were observed using an IX71 inverted microscope (Olympus, Tokyo, Japan). Image acquisition was performed with the Olympus FluoView Software (FV10-ASW Viewer, ver.4.2, https://www.olympus-lifescience.com/en/).

### 4.5. Preparation of Ad-fosARE, WT300 and dl1520 Lysates

To prepare virus lysates, cells were harvested by centrifugation to avoid aspirate non-adherent cells, Ad-*fos*ARE, WT300 and dl1520-infected 293 cells were subjected to three cycles of freezing and thawing. Viral titers infectious units (ifu/mL) were counted using the Adeno-X™ Rapid Titer kit (Clontech Laboratories, Inc., Mountain View, CA, USA) according to the manufacturer’s directions. Virus particles (vp)/mL were determined by a QuickTiter Adenovirus Quantitation kit (Cell Biolabs, San Diego, CA, USA). For in vivo experiments, viral extracts were purified using a Fast-Trap Adenovirus Purification and Concentration kit (Millipore, Billerica, MA, USA) according to the manufacturer’s protocols.

### 4.6. Cytopathic Effect (CPE) Assay and Cell Viability Assay

Cancer and normal cells were seeded on 24-well plates (5 × 10^4^ cells/well). 24 h later, the cells were infected with Ad-*fos*ARE at a multiplicity of infection (MOI) of 1, 10, 50, and 100 ifu/cell and maintained for an additional 7 days. To examine the CPE after combination treatment, cells were treated with paclitaxel and after 4 h treated with Ad-*fos*ARE. Then cells were fixed and stained with Coomassie brilliant blue.

A 2-3-bis [2-methoxy-4-nitro-5-sulfophenyl]-2H-tetrazolium-5-carboxanilide inner salt assay was used to observe the cytolytic activity. Cells were seeded on 96-well plates at 3 × 103 cells/well. 24 h later, the cells were infected with Ad-*fos*ARE at a MOI of 100 ifu/cell. To detect the cell viability after the combination treatment, cells were treated with PTX and after 4 h treated with Ad-*fos*ARE. Cytolytic activity was assessed using an XTT assay on days 1, 3, 5, and 7 with the Cell Proliferation kit II (Roche Diagnostics, Basel, Switzerland) according to the manufacturer’s protocol.

### 4.7. In Vitro Virus Proliferation Assay

Cancer cells and normal cells were seeded at 5 × 10^4^ cells/well 24 h before the infection. Cells were infected with Ad-*fos*ARE, dl1520, or WT300 at an MOI of 10 ifu/cell and medium was not changed after virus infection. After incubation at 37 °C for 48 h, cells were collected, and a virus lysate was prepared as described above. Viral titers (ifu/mL) were determined using the Adeno-X Rapid Titer kit (Clontech Laboratories, Inc., Mountain View, CA, USA).

### 4.8. Western Blot Analysis

Cells were lysed with RIPA buffer (150 mM NaCl; 25 mM Tris-HCl, pH 7.6; 1%Nonidet P-40; 1% sodium deoxycholate; 0.1% SDS) containing protease inhibitors. Equal amount (20 µg) of total protein were separated by 10% sodium dodecyl sulfate-polyacrylamide gel electrophoresis (SDS-PAGE) and transferred onto polyvinylidene difluoride membranes (Millipore, Billerica, MA, USA). Fractionation buffer (10 mM Tris-HCl, pH 7.6, 150 mM NaCl, 1.5 mM MgCl_2_, 0.5% Nonidet P-40, protease inhibitor cocktail) was used to fractionate the cytoplasmic and nuclear proteins, followed by vigorous shaking for 5 min and centrifugation at 12,000 rpm for 30 s. The supernatant was used as the cytoplasmic protein fraction. To assess the accuracy of cell fractionation, cytoplasmic (β-tubulin), and nuclear (lamin B) proteins were detected. For detection of surface protein CAR, the protocol described in Andolazimi et al. was used [23]. Bands were visualized using SuperSignal West Femto Maximum Sensitivity Substrate (Thermo Fisher Scientific, Inc., Waltham, MA, USA). Detailed information about western blot figures can be found at Figures S5–S15.

### 4.9. HuR Depletion

For HuR knockdown, Lipofectamine RNAiMAX (Invitrogen; Thermo Fisher Scientific, Waltham, Massachusetts, USA) was used to transfect HeLa cells with 20 nM each siRNA targeting HuR 1 (5′-AAG UGC AAA GGG UUU GGC UUU UU-3′), HuR 2 (5′-AAU CUU AAG UUU CGU AAG UUA UU-3′) or HuR 3 (5′-UUC GUA AGU UAU UUC CUU UAA UU-3′) with a negative control siRNA (5′-TCT TAA TCG CGT ATA AGG CTT-3′; (Qiagen, Hilden, Germany). After 48 h of transfection, HeLa cells were infected with Ad-*fos*ARE. After 48 h of infection, all cells were scrapped off, and the virus lysate was prepared by three freeze-thaw cycles. Viral titers were detected by using the Adeno-X Rapid Titer kit (Clontech Laboratories, Mountain View, CA, USA) and 293 cells.

For heat shock treatment, HeLa cells were kept at 43 °C for 2 h and immediately infected with Ad-*fos*ARE. Furthermore, after 24 h the infected cells were heat-shocked (2 h) again. Cells were incubated at 48 h after infection, and the viral titers were determined as described above.

### 4.10. RNA Extraction and Quantitative Real-Time RT-PCR

Total RNA was extracted using the RNeasy Mini Kit (250) from QIAGEN and the RNA was subjected to reverse transcription by ReverTra Ace qPCR RT master mix with genomic DNA remover (Toyobo, Osaka, Japan). Quantitative real-time RT-PCR was performed in the DNA Engine Opticon2 (MJ Research, Watertown, MA, USA), and cDNA was amplified using the following primers: Ad5 E1A; 5′-gaa cca ccta ccc ttc acg-3′, 5′-ccg cca aca tta cag agt cg-3′, GAPDH; 5′-atc ctg ggc tac act gag ca-3′, 5′-tgc tgt agc caa att cgt tg-3′. GAPDH was used for normalization. To evaluate the E1A mRNA, HeLa cells were treated with virus and PTX for 24, 48, 72, and 96 h and cellular RNA of each cell was used for quantitative real-time RT-PCR. To estimate the half-life of total E1AmRNA, cells were treated with 5 µL/mL Actinomycin D 60, 120, or 180 min. The total cellular RNA of each cell was used for quantitative real-time RT-PCR.

### 4.11. In Vivo Analysis

Female BALB/c nu/nu mice (five-week-old and 20–22 g) were purchased (Hokudo, Sapporo, Japan) and kept in a specific pathogen-free environment. The temperature was maintained at 26–28 °C, 10 h/14 h light/dark cycle; food and water were given *ad libitum*. Flank tumor was established by injecting HeLa S3 cells (1 × 10^6^ cells/mouse) subcutaneously into the flanks of mice and permitted to grow to ~56 mm in diameter. The mice were assigned into four groups randomly (five per group) and PTX (0.4 mg/100 µL) or vehicle (cremophor EL/dehydrated ethanol/saline) was given by intraperitoneal (i.p) injection. 1 × 10^6^ ifu (100 µL) of Ad-*fos*ARE or the same volume of PBS was injected intratumorally (i.t.) thrice (days 1, 4, and 7) after PTX injection. The perpendicular diameters of the tumors were recorded every 2 or 3 days. Tumor volumes were measured using the following equation: Volume (mm^3^) = A × B^2^ × 0.5 (A is considered the longest diameter, B is considered shortest diameter). The body weight and motor activity of each animal was monitored as indicators of general health and toxicity. The mice were sacrificed by cervical dislocation after 21 days of injection of the virus as the tumors on the control group began to ulcerate. All techniques executed in this study involving animals were performed according to the ethical standards of Animal Care and Use Committee of the Hokkaido University. Sapporo, Japan (Permission number for the animal experiment: 19-0099).

### 4.12. Statistical Analysis

Statistical analysis was performed using one-way ANOVA. Post-hoc multiple comparisons were done by Tukey’s test at a 5% level of significance. All statistical analysis was done by using SPSS 25.0 for Windows (SPSS, Chicago, IL, USA).

## 5. Conclusions

In this manuscript, we have shown that Ad-*fos*ARE, which is a conditionally replicative adenovirus with an ARE component, has high potential as an oncolytic virus. We observed that PTX activates Ad-*fos*ARE through a synergistic manner in cancer cells both in vitro and in vivo. PTX has the potential to enhance cytoplasmic relocalization of HuR, which is required for virus replication. Additionally, PTX upregulates CAR receptor expression and virus entry of the cells. We conclude that these features of PTX are the advantages for Ad-*fos*ARE as an oncolytic virus.

## Supplementary Materials

The supplementary figures are available online at https://www.mdpi.com/2072-6694/12/5/1210/s1, Figure S1: Expression of E1A in Ad-*fos*ARE and WT300-infected cells, Figure S2: Calculation of combination index for Ad-*fos*ARE and PTX, Figure S3: Level of post-translationally modified MTs after combination treatment, Figure S4: The expression of the cleavage of PARP was assessed to detect the cell lysis activity by combination treatment, Figure S5: Detailed information about Figure 1b, Figure S6: Detailed information about Figure 1c, Figure S7: Detailed information about Figure 2a, Figure S8: Detailed information about Figure 2b, Figure S9: Detailed information about Figure 3c, Figure S10: Detailed information about Figure 5a, Figure S11: Detailed information about Figure 5b, Figure S12: Detailed information about Figure 7a, Figure S13: Detailed information about Figure 7d and Figure S3, S14: Detailed information about Figure S1, Figure S15: Detailed information about Figure S4.
