# Supplementary material for: Advantages of Using Paclitaxel in Combination with Oncolytic Adenovirus Utilizing RNA Destabilization Mechanism
Source: Cancers (Basel). 2020 May 12;12(5):1210. doi: 10.3390/cancers12051210 (PMC7281177; doi:10.3390/cancers12051210)
Supplement: Supplementary file 1 [file cancers-12-01210-s001.pdf]

Supplementary Files

Advantages of Using Paclitaxel in Combination with Oncolytic Adenovirus Utilizing RNA Destabilization Mechanism

Elora Hossain, Umma Habiba, Aya Yanagawa-Matsuda, Arefin Alam, Ishraque Ahmed, Mohammad Towfik Alam, Motoaki Yasuda and Fumihiro Higashino

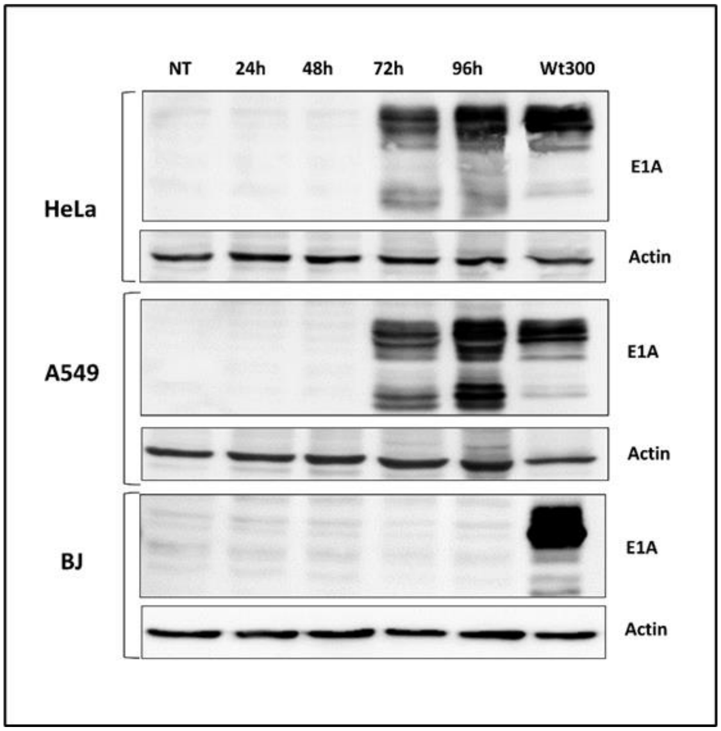

Figure S1. Expression of virus protein in Ad-*fos*ARE and WT300- infected cells. A549, HeLa and BJ cells were infected with both viruses. The Ad-*fos*ARE virus was infected for 24, 48, 72 and 96 h, and WT300 were infected for 24 h. Total cell extracts were prepared, then the expression of early gene E1A and actin was estimated by western blot analysis.

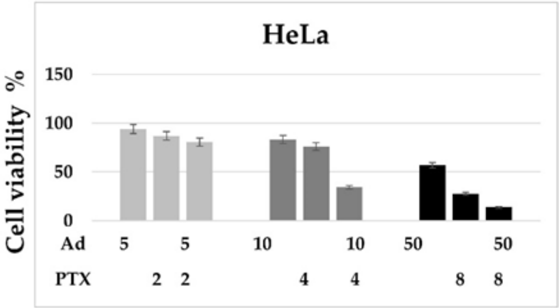

Dose PTX nM	Ad-*fos*ARE ifu/cell	Effect	CI
**2.0**	5.0	0.6	0.67835
**4.0**	10.0	0.22	0.50583
**8.0**	50.0	0.13	0.85878

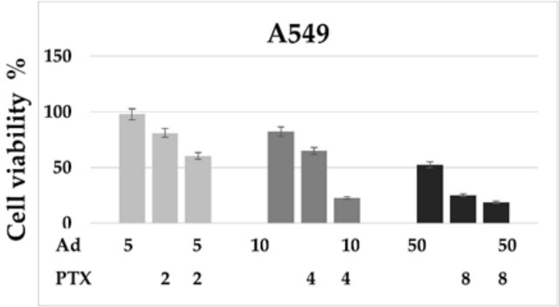

Dose PTX nM	Ad-*fos*ARE ifu/cell	Effect	CI
**8.0**	50.0	0.81	0.97063
**4.0**	10.0	0.77	0.52767
**2.0**	5.0	0.6	0.40865

Figure S2. Calculation of combination index for Ad-*fos*ARE and PTX. HeLa and A549 cells were transfected with 3 different dosages of Ad-*fos*ARE, PTX, and combination. CIs of Ad-*fos*ARE or PTX or in combination with were calculated using Compusyn and plotted against the percentage of cell death resulting from combined therapy. CI of < 0.9 indicates synergy, CI between 0.9 and 1.1is addictive, and CI of > 1.1 indicates antagonism.

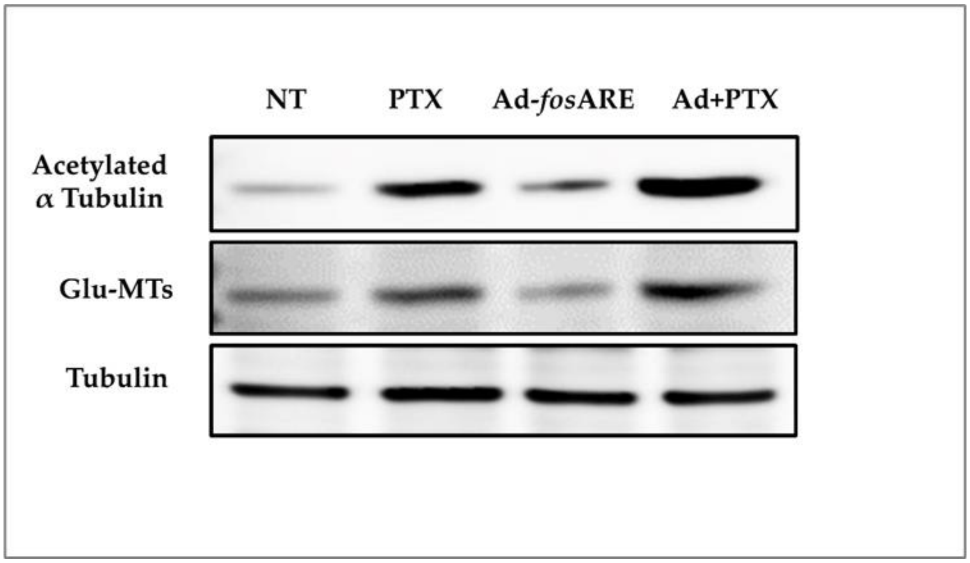

Figure S3. Level of posttranslationally modified MTs after combination treatment. To determined posttranslationally modified tubulin, HeLa cells were treated with Ad, PTX and combination. The expression of acetylated and Glu-MTs was quantified by densitometry and the values were normalized to non-infected conditions.

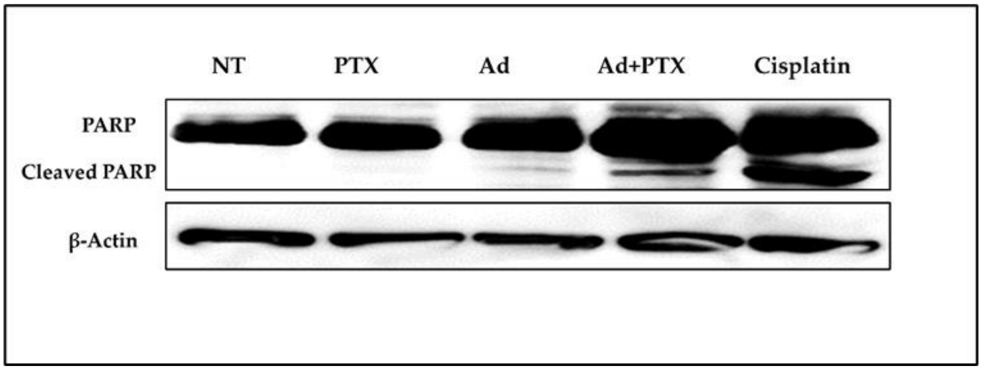

Figure S4. The expression of the cleavage of PARP was assessed to detect the cell lysis activity by combination treatment. HeLa cells were infected with the virus, PTX and combination and whole-cell lysate were subjected to SDS-PAGE gel, and the cisplatin-treated lysate was used as a positive control.

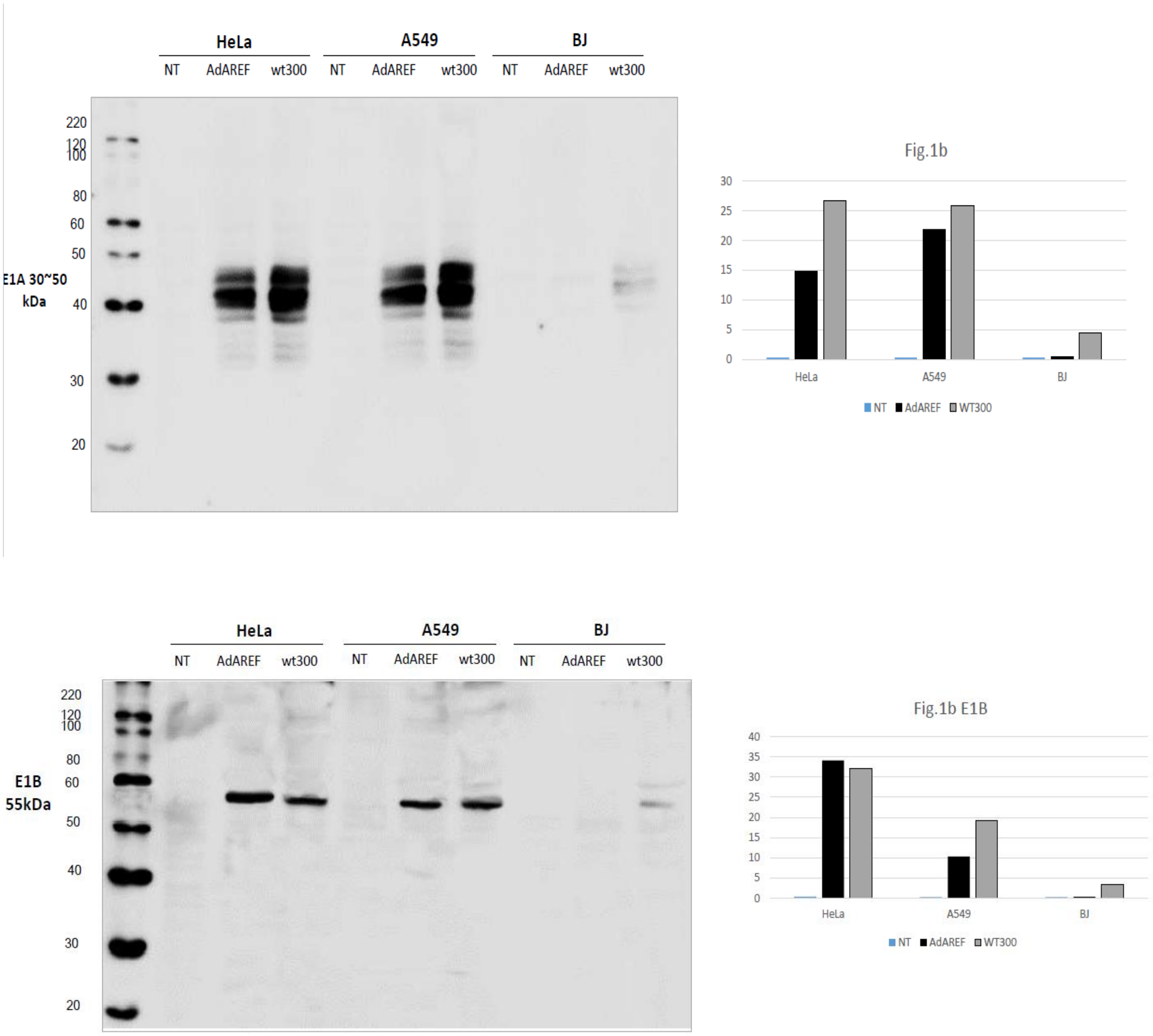

Figure S5. Detailed information about Figure 1b.

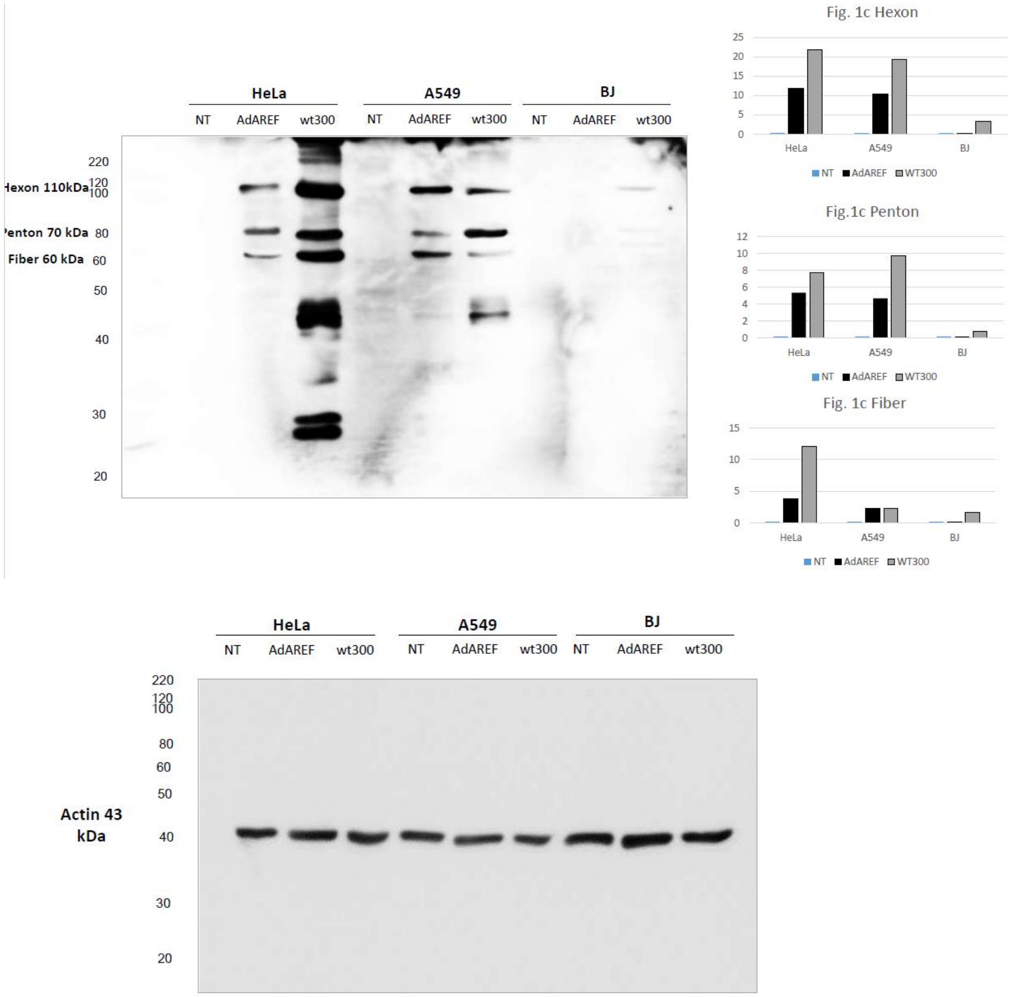

Figure S6. Detailed information about Figure 1c.

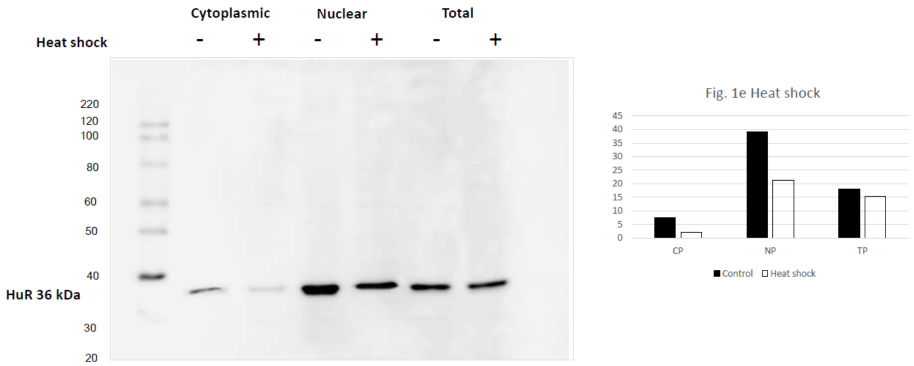

Figure S7. Detailed information about Figure 2a.

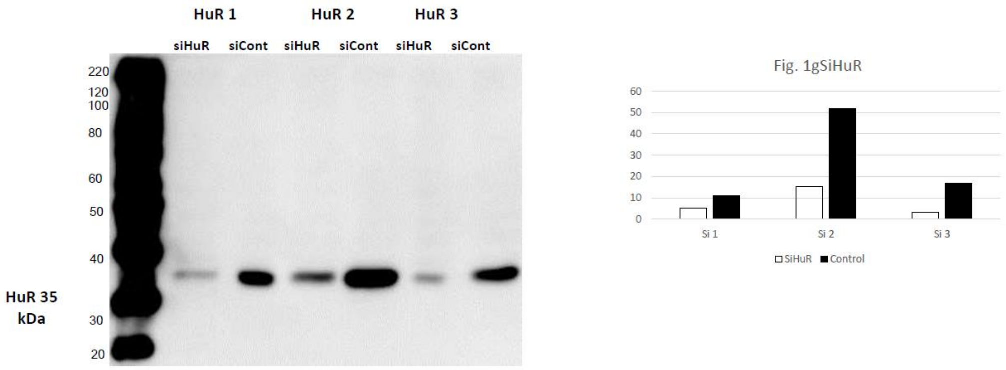

Figure S8. Detailed information about Figure 2b.

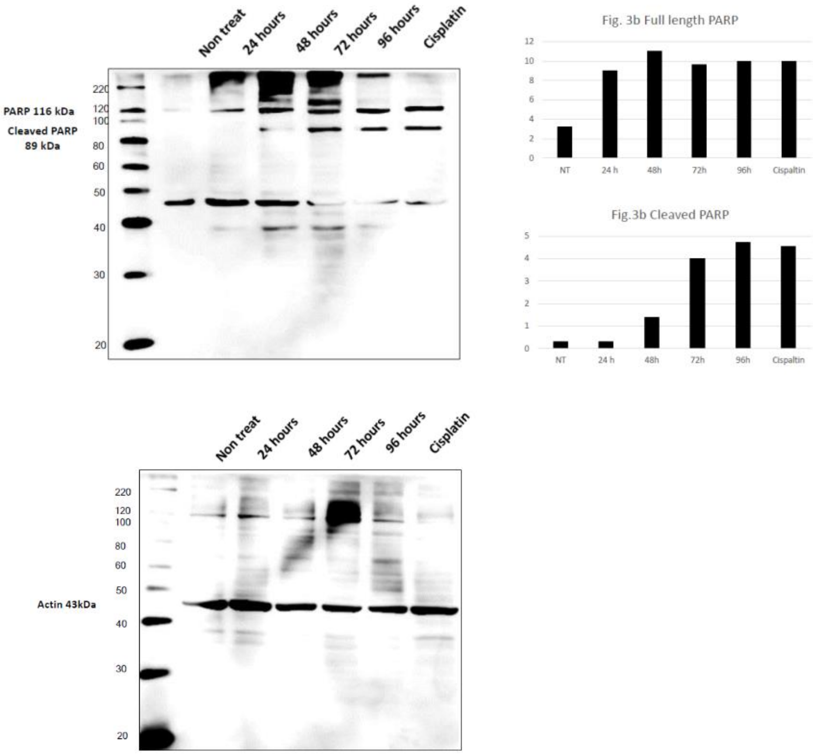

Figure S9. Detailed information about Figure 3c.

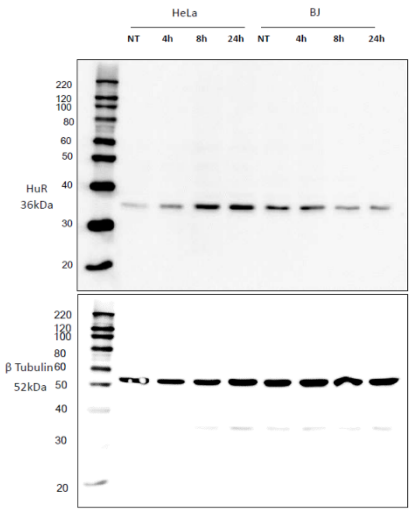
HeLa
BJ
NT
4h
8h
24h
NT
4h
8h
24h
220
120
100
80
60
50
40
30
20
HuR
36kDa
β Tubulin
52kDa

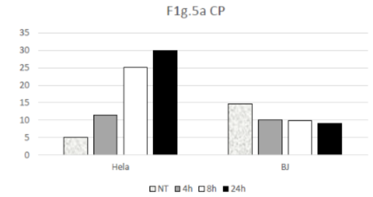
F1g.5a CP
35
30
25
20
15
10
5
0
Hela
BJ
NT
4h
8h
24h

5a

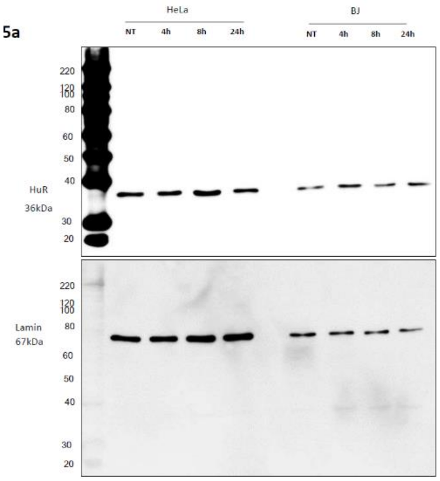
HeLa
BJ
NT
4h
8h
24h
NT
4h
8h
24h
220
120
100
80
60
50
40
30
20
HuR
36kDa
Lamin
67kDa

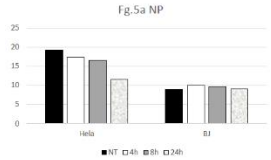
Fg.5a NP
25
20
15
10
5
0
Hela
BJ
NT
4h
8h
24h

10

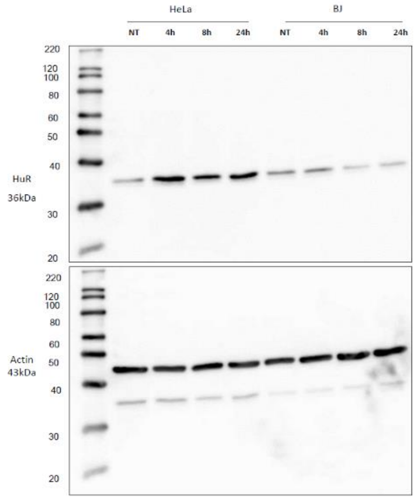
HeLa
BJ
NT
4h
8h
24h
NT
4h
8h
24h
220
120
100
80
60
50
40
30
20
HuR
36kDa
Actin
43kDa

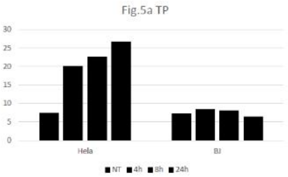
Fig.5a TP
30
25
20
15
10
5
0
Hela
BJ
NT
4h
8h
24h

11

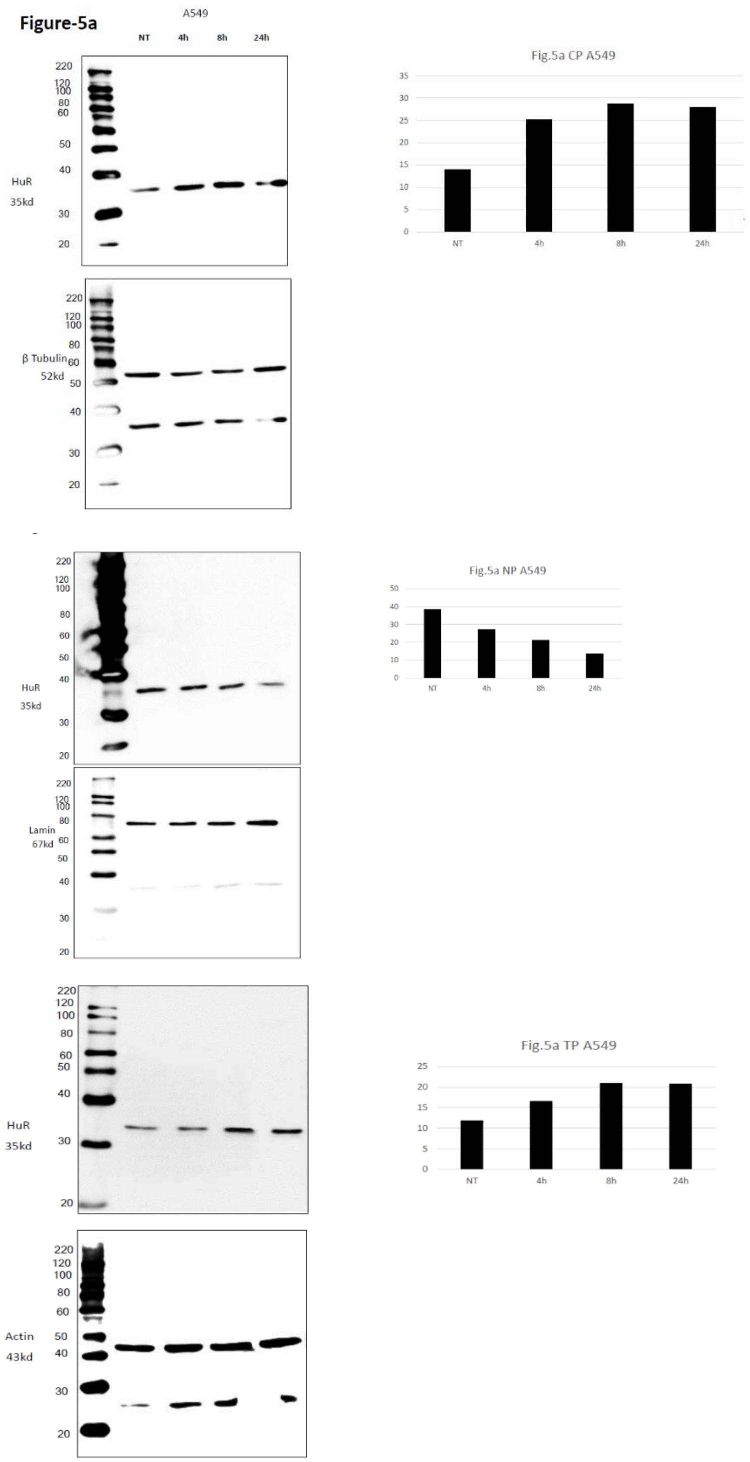

Figure S10. Detailed information about Figure 5a.

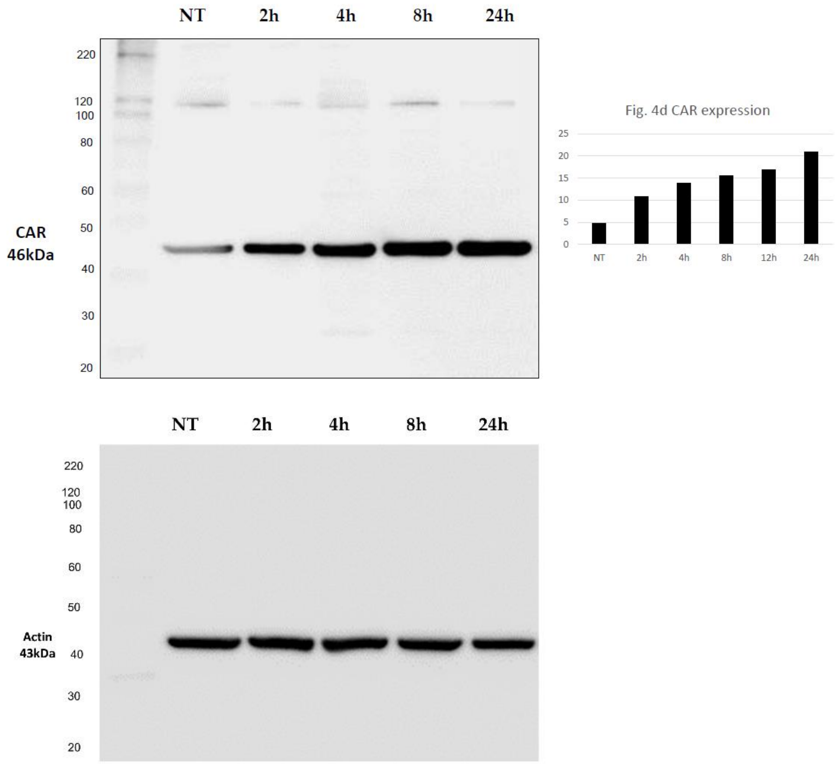

Figure S11. Detailed information about Figure 5b.

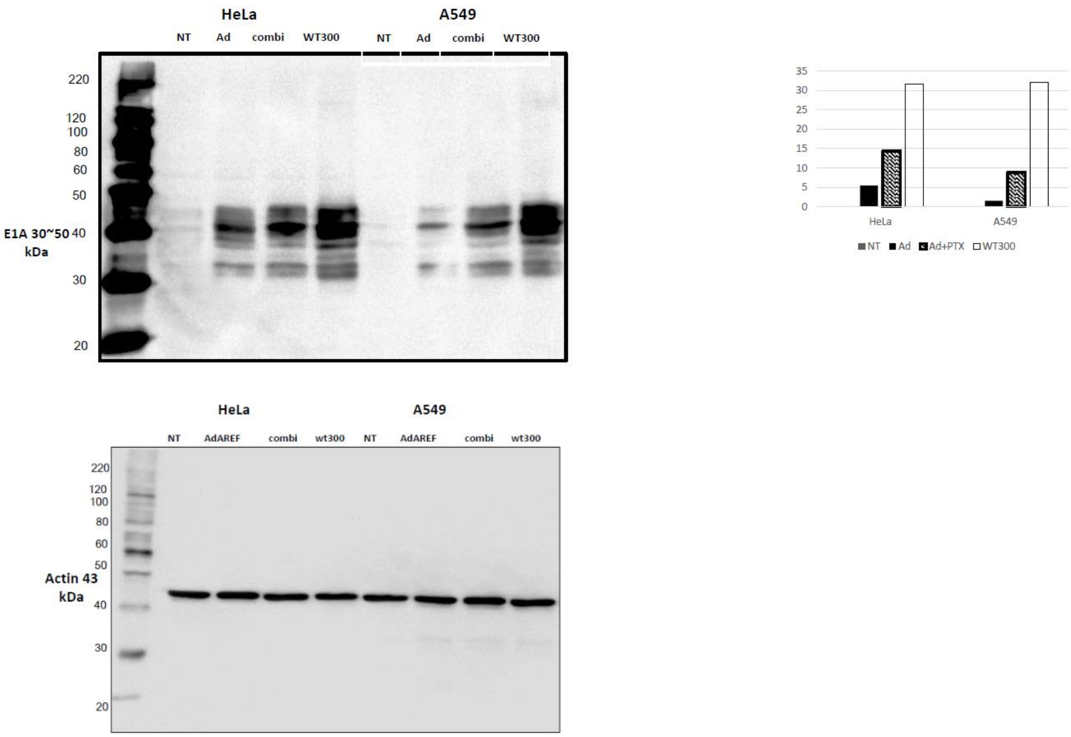

Figure S12. Detailed information about Figure 7a.

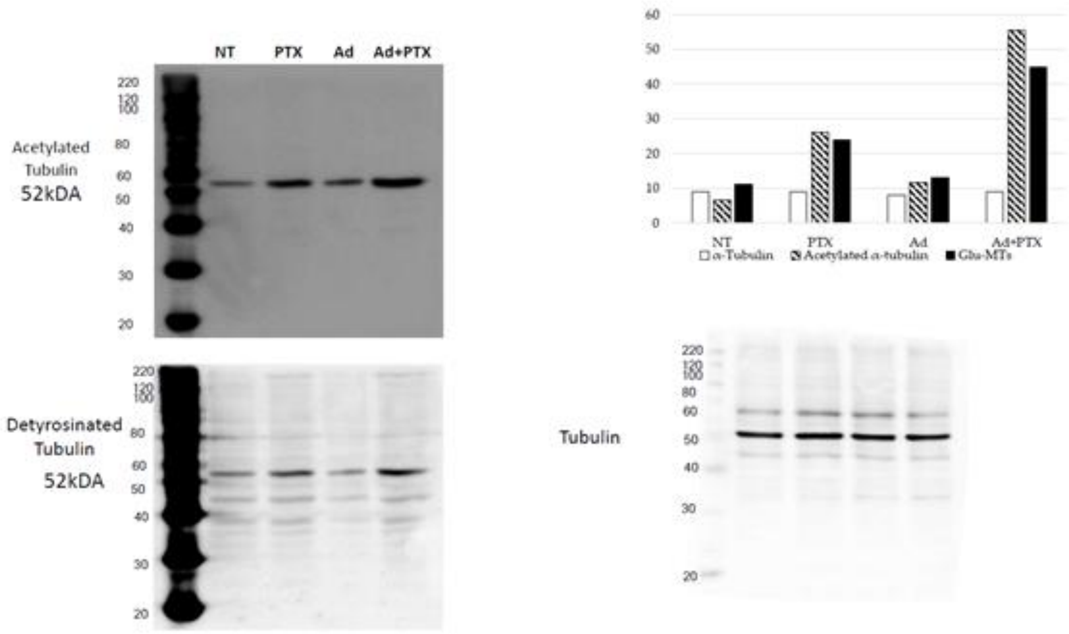

Figure S13. Detailed information about Figure 7d.

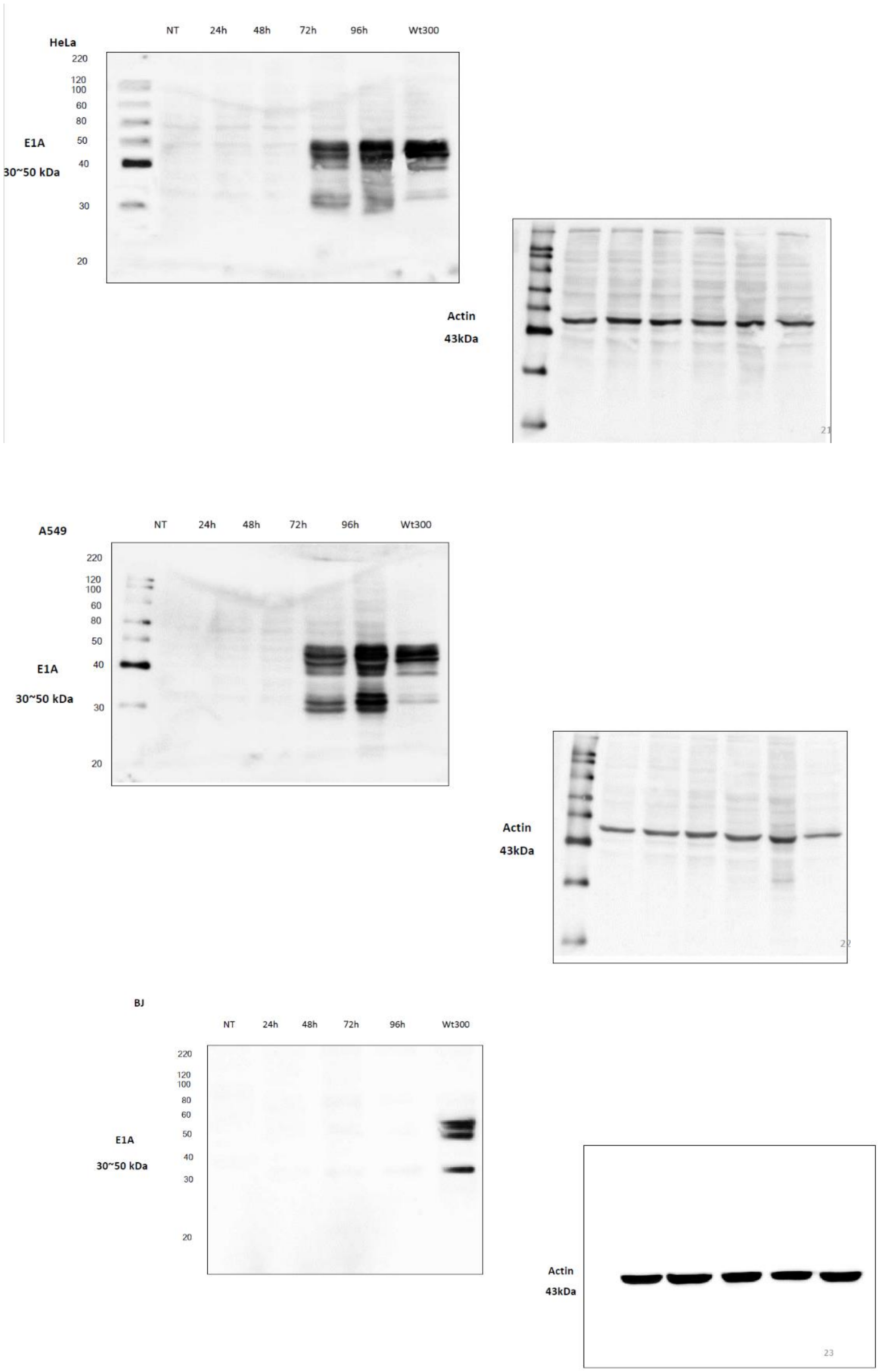

Figure S14. Detailed information about Figure S1.

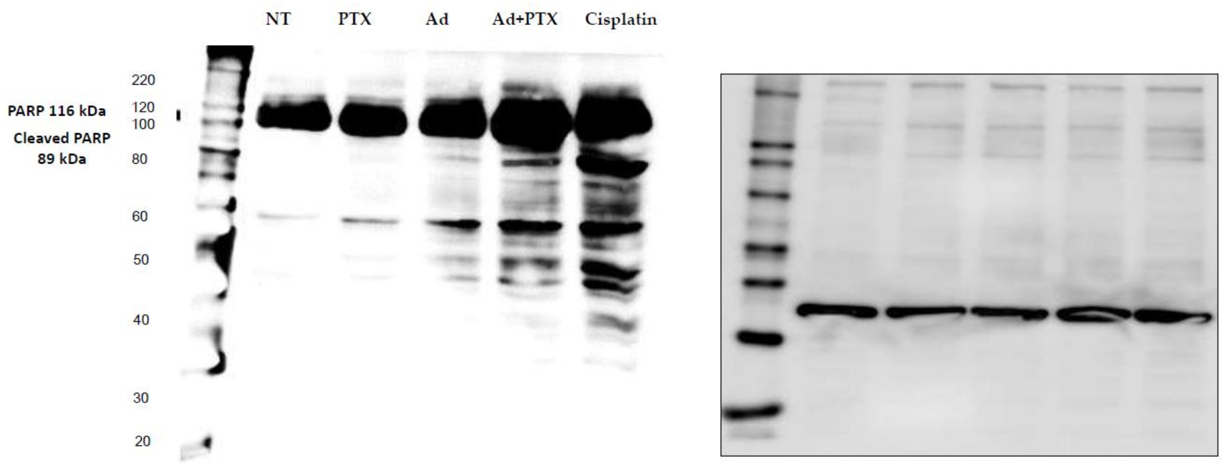

Figure S15. Detailed information about Figure S4.
